# Climate resilience of dry season cereals in India

**DOI:** 10.1038/s41598-023-37109-w

**Published:** 2023-06-20

**Authors:** Ruth DeFries, Shefang Liang, Ashwini Chhatre, Kyle Frankel Davis, Subimal Ghosh, Narasimha D. Rao, Deepti Singh

**Affiliations:** 1 Department of Ecology, Evolution, and Environmental Biology, Columbia University, New York, 10027 USA; 2 Climate School, Columbia University, New York, 10027 USA; 3 Institute of Agricultural Resources and Regional Planning, Chinese Academy of Agricultural Sciences, Beijing, 100081 China; 4 Indian School of Business, Hyderabad, 500111 India; 5 Department of Geography and Spatial Sciences, University of Delaware, Newark, DE 19716 USA; 6 Department of Plant and Soil Sciences, University of Delaware, Newark, DE 19716 USA; 7 Department of Civil Engineering, Indian Institute of Technology Bombay, Powai, MH India; 8 Yale School of the Environment, Yale University, New Haven, CT 06520 USA; 9 International Institute for Applied Systems Analysis, Laxenburg, Austria; 10 School of the Environment, Washington State University, Vancouver, WA 98686 USA

**Keywords:** Climate sciences, Ecology

## Abstract

India is the world’s second largest producer of wheat, with more than 40% increase in production since 2000. Increasing temperatures raise concerns about wheat’s sensitivity to heat. Traditionally-grown sorghum is an alternative *rabi* (winter season) cereal, but area under sorghum production has declined more than 20% since 2000. We examine sensitivity of wheat and sorghum yields to historical temperature and compare water requirements in districts where both cereals are cultivated. Wheat yields are sensitive to increases in maximum daily temperature in multiple stages of the growing season, while sorghum does not display the same sensitivity. Crop water requirements (mm) are 1.4 times greater for wheat than sorghum, mainly due to extension of its growing season into summer. However, water footprints (m^3^ per ton) are approximately 15% less for wheat due to its higher yields. Sensitivity to future climate projections, without changes in management, suggests 5% decline in wheat yields and 12% increase in water footprints by 2040, compared with 4% increase in water footprint for sorghum. On balance, sorghum provides a climate-resilient alternative to wheat for expansion in *rabi* cereals. However, yields need to increase to make sorghum competitive for farmer profits and efficient use of land to provide nutrients.

## Introduction

Cereals are the backbone of the present-day human diet, with wheat providing a fifth of dietary calories worldwide^1^. India is the world’s second largest producer of wheat after China. The vast majority (> 99% for 2015–2019) of wheat produced in India is consumed domestically by its population of 1.38 billion people, but low-income countries including Bangladesh, Nepal, Afghanistan, Sri Lanka, and Somalia rely on exports from India^2^.

Sensitivity of wheat to temperature is widely documented^3,4^. Increases above optimum temperatures adversely affect crop phenology, growth, and development. Optimum temperatures vary by growth stages, ranging from 17 °C for root growth to 26 °C for grain filling. In India, many studies have combined crop models with climate projections to assess impacts on wheat yields and identify possible adaptation strategies, e.g.^5–8^. These studies indicate that adjustment in sowing time, heat-tolerant varieties, residue to conserve moisture, and crop switching can reduce the impact of increasing temperatures^9,10^.

With the 2022 Ukraine conflict restricting global wheat supplies and creating a crisis from surging prices, the government of India announced plans to share its bumper harvest with vulnerable countries. Subsequently, a heat wave in March 2022 severely affected the harvest, and the government banned exports in May amidst rising domestic prices^11^, though continued to provide wheat to the most vulnerable countries as humanitarian assistance^12^. While this incident may be particular to the unusual confluence of a conflict in eastern Europe with an extreme climate event in India, it illustrates the vulnerability for India and importing countries from reliance on a temperature-sensitive staple.

Wheat in India is the predominant cereal produced in the post-monsoon dry winter season (known as the *rabi* season), often in rotation with rice grown in the monsoon (*kharif*) season. Sorghum, the cereal with the fifth highest production in the world after maize, rice, wheat, and barley, is produced in India in both *rabi* and *kharif* seasons. Sorghum (*jowar* in Hindi) is a critically important staple in semi-arid and arid regions in Sub Saharan Africa and South Asia for millions of small and marginal farmers with mixed cropping-livestock systems. *Kharif* sorghum is used mainly for animal feed and industry. *Rabi* sorghum is higher quality, consumed directly in porridges and bread, and used for fodder^13^. In contrast, sorghum grown in industrialized countries is used mainly for commercial animal feed and industrial processes.

As a C4 grass with dense root masses, sorghum is drought resistant and hardy in harsh growing conditions, although not immune to the impacts of climate change^14,15^. A C4 plant’s leaf anatomy allows it to keep its stomata closed and retain water during photosynthesis. C4 crops, including maize, millets, and sorghum, are water efficient, tolerant to low moisture environments, adaptable to a wide range of soil conditions, and reach maturity in relatively short time periods. Wheat, a C3 crop, is less water efficient and more temperature sensitive^16^. Because irrigation and/or soil moisture is a necessity for *rabi* production and most parts of India are water stressed, a crop’s water requirement is a key consideration for climate-resilience in the *rabi* season.

Wheat, along with inputs of irrigation, fertilizer, agro-chemicals and cultivars, has been the mainstay of India’s ability to increase per capita production of calories and outpace population growth since the Green Revolution^17^. *Rabi* production continues to be the predominant mode for increasing cereal production^18^. Sorghum has not benefited from the same investments and improved varieties as wheat due the emphasis on high-yielding hybrid crops and monoculture^19^.

Many studies highlight the need for increasing production of alternative C4 cereals, such as millets and sorghum, for resilience to a changing climate^20^. In the early 1960s, prior to the Green Revolution, C4 cereals comprised approximately 35% of rural India’s per capita consumption of cereals with 15% wheat and the remainder rice. By 2012, the proportions reversed to approximately 5% for C4 cereals and 38% wheat^21^.

C4 *kharif* cereals (pearl millet, finger millet and sorghum) provide advantages over rice, the overwhelmingly predominant *kharif* cereal, across multiple dimensions including human nutrition, water and energy use, and greenhouse gas reductions. The Government of India has recognized “nutri-cereals”, introduced millets in the public distribution system, and promoted the 2023 International Year of Millets^22^. Unlike the nutritional benefits of C4 *kharif* cereals compared to rice, wheat and sorghum are roughly comparable in terms of nutrition content (Table A4 in^23^) but potentially different in terms of climate resilience and water requirements.

In this paper, we focus on the two main *rabi* cereals in India, wheat and sorghum, to highlight the need for climate-smart agriculture specifically in that season. Specifically, this paper: examines historical patterns and trends of *rabi* cereal production in India; compares temperature sensitivity and water requirements for the two cereals; and assesses the sensitivity of yields and water requirements of both cereals to increasing temperature in the future.

## Results

### Trends in *rabi* cereals

**Figure 1:**
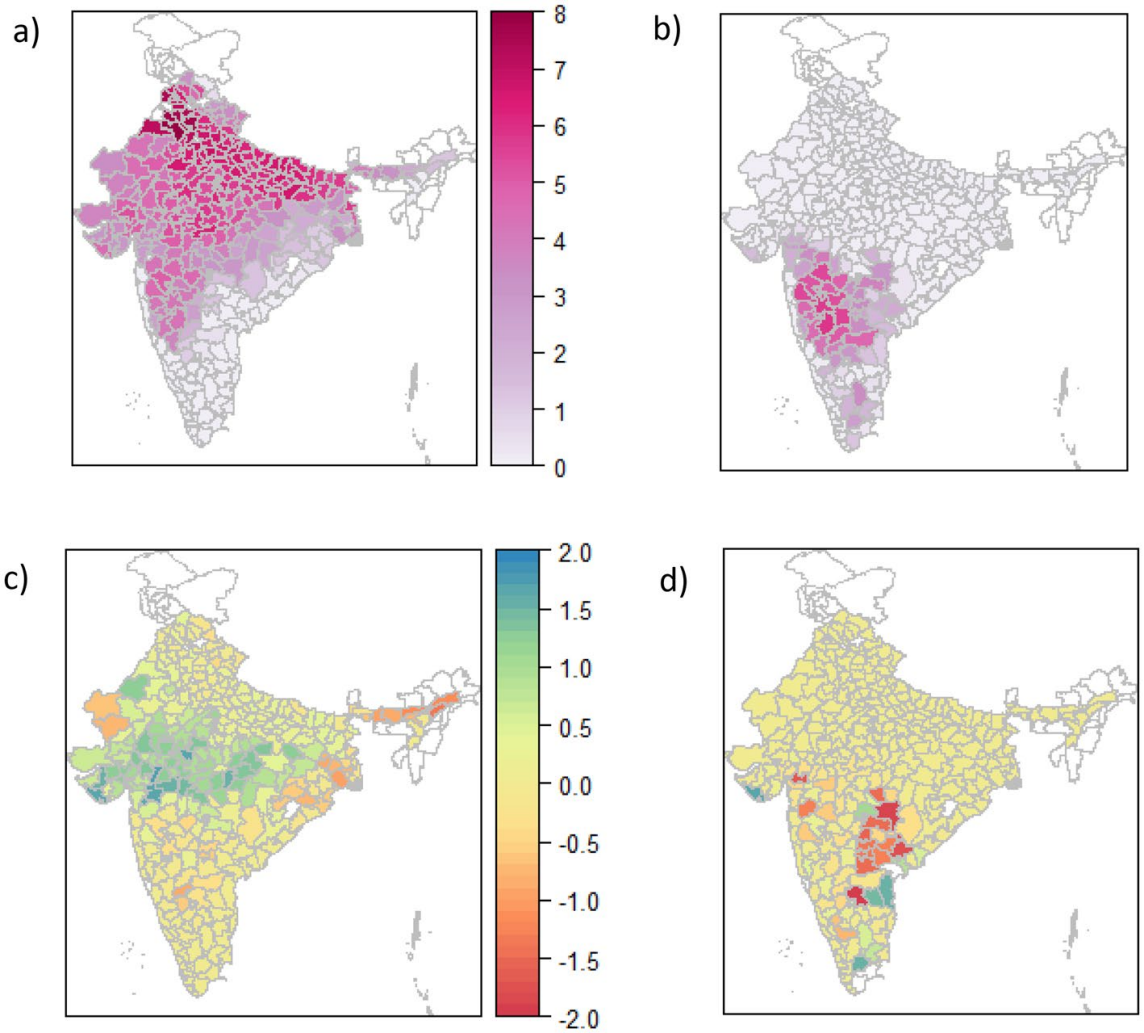
Production by district average for 1998–2002 (1000 tonnes/year in units of log + 1) for wheat (**a**) and sorghum (**b**) and difference from 1998–2002 to 2012–2017 for wheat (**c**) and sorghum (**d**). Data from Ref.^28^.

The trend of increasing wheat production in India that began with the Green Revolution in the 1960s continues until the present. Total wheat production in the country increased 42% since the turn of the century (average 1998–2002 relative to average 2012–2017), due both to increases in yield (26%) and increase in area (17%). While the northwest is the major center of production, area under wheat production has expanded southward into central, warmer and more arid parts of the country (Fig. 1).

*Rabi* sorghum shows a contrasting trend. Total sorghum production, which is concentrated in the semi-arid central parts of the country, has declined by 5% in the same time period. This decline is despite an 37% increase in yields and is attributable to a 21% loss in area under production.

**Figure 2:**
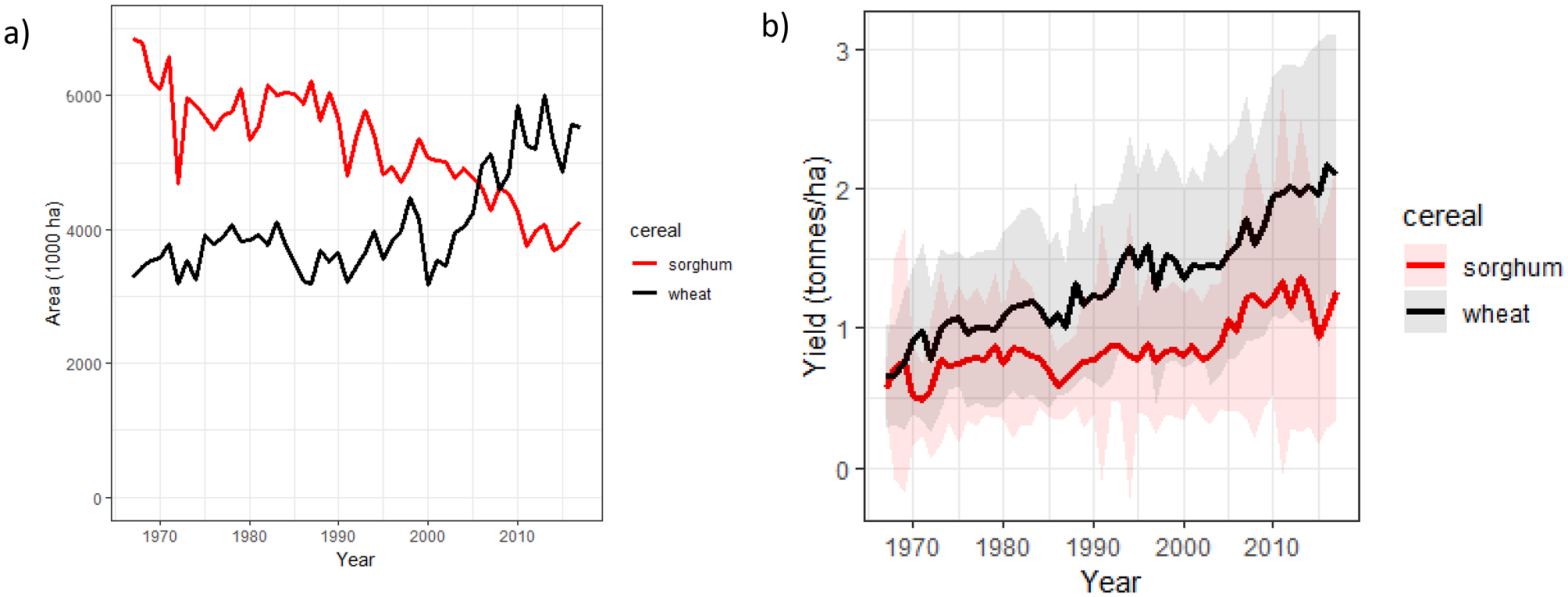
Area (left) and yields (right) from 1967 to 2017 for wheat and sorghum in the 101 districts (historical boundaries) where both cereals are grown. Error envelopes are standard deviations. Data from Ref.^28^.

In the common districts where both sorghum and wheat are cultivated (Supplemental Fig. 1), wheat area has risen while sorghum area has declined, particularly since the turn of the century when wheat surpassed sorghum (Fig. 2). The common districts constituted 18% of all wheat production and 98% of all sorghum production in the country in the 2012–2017 timeframe. Yields for both cereals have increased in these districts since the turn of the century, but wheat yields remain higher (2.04 ± 0.09 (sd across districts) and 1.17 ± 0.15 tonnes/ha for wheat and sorghum respectively for 2012–2017).

With increasing demand for wheat and the trend towards *rabi* cereal production^18^, wheat is likely to continue to expand into warmer, semi-arid regions of the country. Exposure to higher temperatures and water stress elevates the importance of the resilience of *rabi* cereals to climate change.

### Sensitivity of yields to temperature

Model results show significant negative associations between wheat yields and median of maximum daily temperature in both stage 2 (Nov 7 to Feb 13) (p = 0.006) and stage 3 (Feb 14 to April 11) (p = 0.008) in the common districts where both sorghum and wheat are cultivated, with higher sensitivity in stage 1. One degree C increase in median maximum daily temperature in stage 1 and stage 2 is associated with reductions of 0.04 and 0.03 tonnes/ha respectively, for a total reduction of approximately 3.4% of yield. Precipitation is not significant in either stage, an unsurprising result because *rabi* production depends largely on irrigation or residual soil moisture from the monsoon (Table 1).

**Table 1:** Model results from mixed linear models for wheat and *rabi* sorghum yields from 1967 to 2018 for districts where both wheat and sorghum are grown.

Variable	Unit	Wheat model	Sorghum model
Dependent variable	tonnes/ha/year (× 10^–3^)	Wheat yield ($${Y}_{s)}$$	Sorghum yield $${(Y}_{w})$$
Predictor variables			
${T}_{stage1}$	Median maximum daily temperature (C) from Sept-19 to Nov-06	–	27.27 (11.62)^#^
${T}_{stage2}$	Median maximum daily temperature (C) from Nov-07 to Feb-13	− 40.58 (14.79)*	0.41 (14.67)
${P}_{stage2}$	Total precipitation (mm) from Nov-07 to Feb-13	− 0.21 (0.23)	− 0.11 (0.22)
${T}_{stage3}$	Median maximum daily temperature (C) from Feb-14 to Apr-11	− 30.63 (11.62)*	–
${P}_{stage3}$	Total precipitation (mm) from Feb-14 to Apr-11	− 1.26 (0.51)	–
${S}_{1}$	Average proportion of sand (%)	30.77 (13.74)^#^	− 47.34 (12.75)***
${S}_{2}$	Average proportion of silt (%)	− 8.13 (18.94)	− 40.06 (17.22)*
n districts		101	101
n years		51	51
n observations		2788	2788
Conditional R2		0.79	0.74
Marginal R2		0.03	0.09

Standard error in parentheses. ***p < 0.001, **p < 0.005, *p < 0.01, ^#^p < 0.05. Data sources listed in Supplemental Table 5.

Sorghum yield is significantly (p = 0.019) associated with temperature in stage 1 (Sept 19 to Nov 6) but with a positive coefficient. A possible explanation is that higher temperature, which is associated with less precipitation, correlates with less cloudiness leading to more radiation that increases yields. Only maximum daily temperature is included in the sorghum model for stage 1 due to co-linearity between temperature and precipitation variables (see “Methods” section). Neither temperature nor precipitation is significant in the sorghum model in stage 2.

In summary, based on significance of variables in the generalized linear mixed model, the significantly negative association between maximum temperature and yields at the district level from 1967 to 2017 is in line with other studies highlighting the temperature sensitivity of wheat in all growth stages. Sorghum does not show the same sensitivity with the historic data. Other factors indirectly associated with temperature, such as precipitation and radiation, may be more of an influence on sorghum yields than temperature per se. In contrast to wheat, soil characteristics are more consequential for sorghum yields in the historical data, possibly because farmers are more likely to grow sorghum on sandier, less productive soils (Table 1).

**Figure 3:**
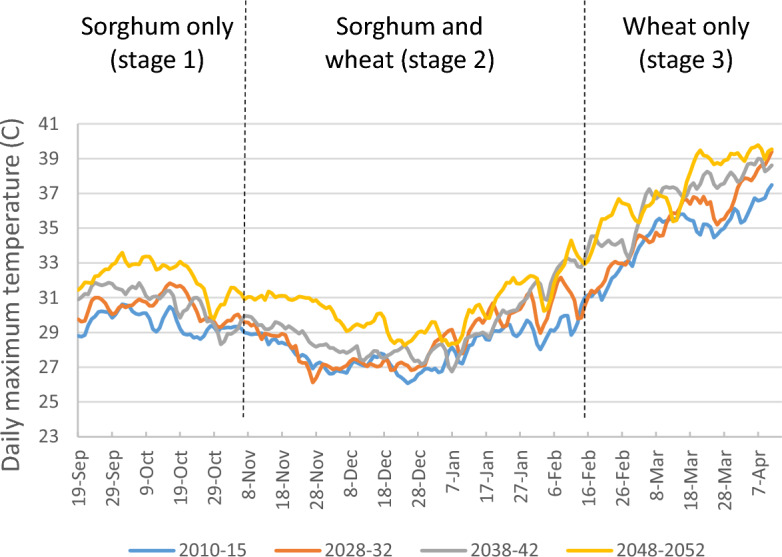
Projections for daily maximum temperature averaged across time periods (2010–2015; 2028–2032; 2038–2042; 2048–2052) and common districts where both wheat and sorghum are grown from the CMIP6 CNRM-ESM2-1 climate model.

Future projections for maximum daily temperature during the *rabi* season for the highest emissions scenario (SSP8.5) from the CNRM-ESM2-1 climate model illustrate the variable but upward trend throughout all stages in the wheat and sorghum growing season in the common districts where both wheat and sorghum are grown (Fig. 3, Supplemental Table 1). Temperatures are highest after the sorghum harvest at the end of the wheat growing season, the time period corresponding to the heat wave that affected the 2022 harvest. The alternate climate model, CNRM-CM6-1-HR, shows similar trends and comparable projections for total precipitation in the 3 stages (Supplemental Fig. 2).

**Figure 4:**
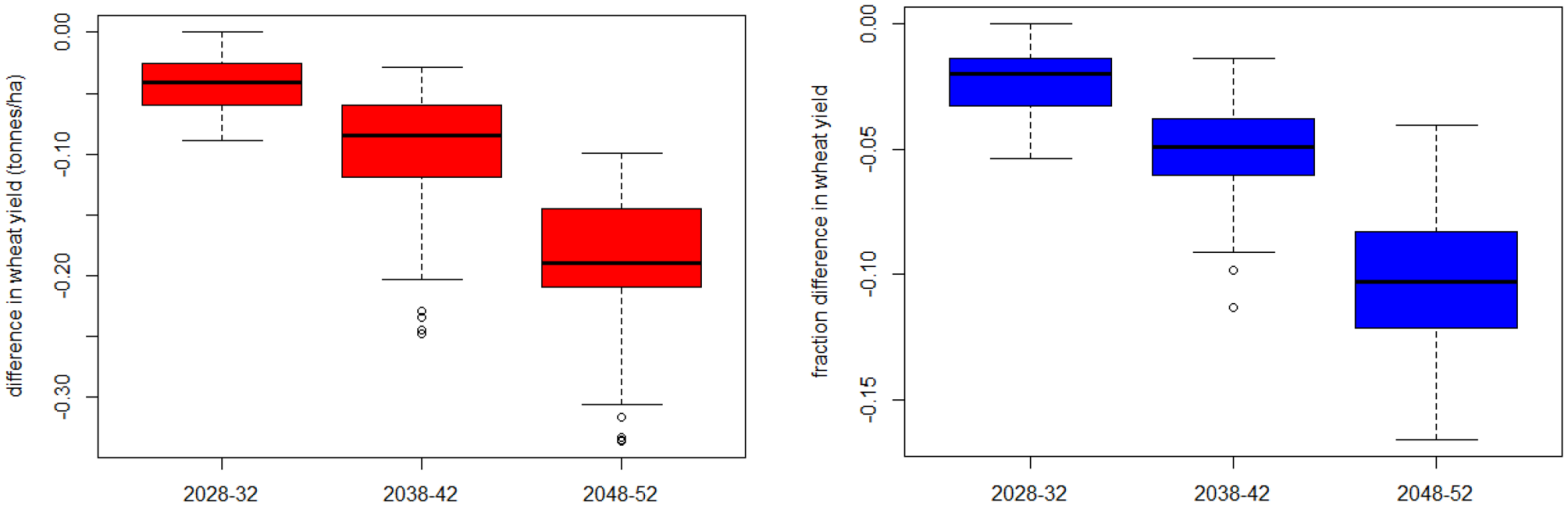
Sensitivity of wheat yields to projections of maximum daily temperature from the CMIP6 CNRM-ESM2-1 climate model in Fig. 3.

Based on the wheat model for these climate projections, the sensitivity of wheat yields to maximum daily temperature shows a difference in average yields across the districts by − 0.04 ± 0.02 (sd), − 0.09 ± 0.05, and − 0.18 ± 0.05 tonnes/ha by 2028–2032, 2038–2042, and 2048–2052 respectively relative to 2010–2015 baseline in the common districts (Fig. 4). These differences in yield correspond to percentage differences of − 2.4 ± 1.3, − 5.0 ± 1.8, and − 10.3 ± 2.8%. The sensitivities to climate projections from the alternate climate model, CNRM-CM6-1-HR, are higher but within the same range for the 2028–2032 time-period (− 5.3 vs − 2.4%) and 2038–2042 (− 7.9 vs − 5.0%) but lower for 2048–2052 (− 9.7 vs − 10.3%) (Supplemental Fig. 3).

These projections are intended to assess the sensitivity to projected climate change and not to predict actual yields that will occur in the future. They do not account for future yield increases from improved varieties, management, direct effects of atmospheric carbon dioxide and other factors responsible for changes in yield in the last decades, which will likely continue into the future. Further, they do not account for the uncertainty in climate projections from differences between climate models, natural climate variability, and future emissions trajectories.

### Water requirements

**Figure 5:**
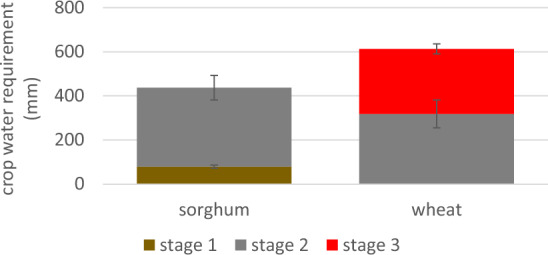
Mean crop water requirements (mm) for rabi sorghum and wheat for common districts averaged for 2010–2015. All parameters for the Penmen–Monteith equation obtained from the CMIP6 CNRM-ESM2-1 climate model^38^ for cwr in stage 1 (Sept 19–Nov 6), stage 2 (Nov 7–Feb 13), and stage 3 (Feb 14–April 11). Error envelope is 95% confidence interval. Supplemental Table 7 “cwr future” provides cwr estimates from the alternative climate model and for future climate projections for both models.

Consistent with expectations for water use by C3 and C4 crops, the amount of water (mm) required to keep the plant from experiencing water stress averages 437.35 ± 12.26 and 613.60 ± 12.48 across the common districts for sorghum and wheat respectively for the 2010–2015 baseline time period (Fig. 5). The water requirement for sorghum is slightly higher than wheat in the overlapping stage (Nov 7 to Feb 13). However, the water requirement during the final stage for wheat, the hottest time of year, is almost as demanding as the overlapping stage 2 for a fewer number of days (99 vs 57 days).

With projected future temperatures from CMIP6 CNRM-ESM2-1, estimates for total crop water requirements increase for both cereals, e.g. from 437.35 in 2010–2015 by approximately 6% in 2028–2032 for sorghum and from 613.60 in 2010–2015 by approximately 9% for wheat. Total crop water requirements for wheat remain between 38 and 44% higher than sorghum throughout all time periods. The overall trend is similar with estimates using climate parameters from CNRM-CM6-HR-1 model (Supplementary Table 2).

**Figure 6:**
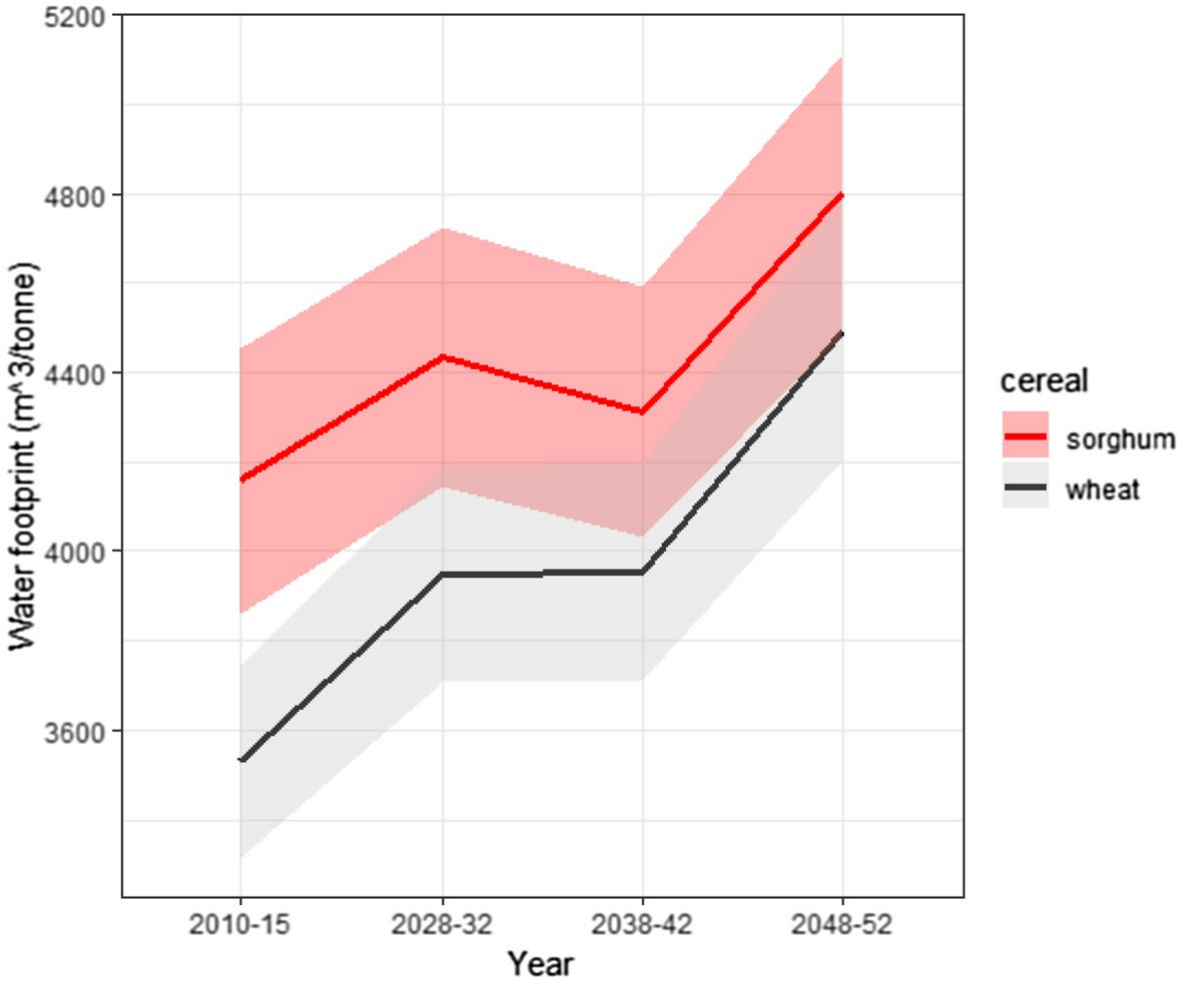
Water footprint (m^3^/tonne) average across common district based on crop water requirements and predicted yields. See Supplemental Fig. 4 for results using climate parameters from CNRM-CM6-HR-1.

Estimates for water footprints (volume of water per unit of production) for the two *rabi* cereals are counter to the crop water requirements. Due to the difference in yields, wheat’s water footprint is 15% lower than for sorghum averaged across the common districts for 2010–2015. Accounting for both wheat yield decreases from climate change and increased water requirements (assuming no changes in future sorghum yields based on results in Table 1) wheat’s water footprint remains lower than sorghum for all time periods but the gap closes from approximately 15% to 6% by 2048–2052 (Fig. 6).

From the perspective of a water supplier, water requirements for sorghum are satisfied with less water than for wheat. From the perspective of a farmer, wheat’s lower water footprint maximizes production for the amount of water available, even with the reduction in yield from higher temperatures. Increases in sorghum yields are needed to reduce the water footprint and benefit from the lower water requirement and less sensitivity to increasing temperatures.

## Discussion

Trends in India towards increasing cereal production in the *rabi* season and expansion of wheat cultivation from the northwest into hotter parts of the country indicate a need to consider climate-resilient strategies for cereals grown in the *rabi* season. Calls by scientists and activists for diversifying *kharif* cereals has been met with increased government focus on millets for nutrition and climate resilience. Similarly, a focus on stemming the contraction of *rabi* sorghum could improve climate resilience and food security for small and marginal farmers in semi-arid sorghum-growing regions as temperatures continue to rise. The higher quality of *rabi* sorghum (used for human consumption) compared with *kharif* sorghum (used for fodder and industry) further highlights the importance for food security.

Compared with the clear advantage of *kharif* millets over rice from a nutritional point of view^20,21^, wheat and sorghum do not present such a stark nutritional contrast. However, the sensitivity to increasing temperature and lower water requirements can be key elements for farmers, particularly with improved management practices to increase yields through high-yielding varieties, optimum planting, and other measures^24^. In addition, the strong negative association between sandy soil and yields for *rabi* sorghum (Table 1) suggests improvements in yield with sorghum cultivation on soils with high water holding capacity. For wheat farmers, the timing of the growing season to minimize exposure in the hottest time of the year as well as drought-resistant varieties could reduce the impacts of future heat waves such as in March 2022.

The trend in the last several decades has been decreasing water footprints from cereal crops due to both increasing yields and declining crop water requirements^18^. Observations identify declining evapotranspiration throughout the country since the late 1960s, attributable to positive trends in relative humidity and negative trends in wind speed and solar radiation^25^. To the extent that the CMIP6 climate models used in this study accurately project these variables into the future, results from this paper suggest that declining evapotranspiration trends might be reversed due to increases in future temperatures in response to continued emissions of greenhouse gases.

This paper focuses on empirical relationships between temperature and yields for *rabi* cereals and uses existing methods to estimate water requirements. Many important factors are not included. First and foremost, management and adaptation strategies can offset impacts of climate change on yield to varying degrees^15^. Second, increasing carbon dioxide concentrations directly affect C3 (wheat) and C4 (sorghum) plants differently. Rates of photosynthesis and water use efficiency are more responsive to increasing CO_2_ concentration than C4 plants, but C3 plants suffer more declines in nutrient content than C4 plants^26,27^. Pests, disease, and extreme events such as hail storms are not incorporated in projections of future yield. Finally, the projected impacts on yields assume the same relationship between temperature and precipitation and yields and might not capture non-linear relationships beyond the range of historical data used to construct the models. In the near-term (2028–2052) projected climate data used in this analysis to test sensitivity to yields, future district-level median of daily maximum temperature over the study region is 0.2 °C higher than the historical range in stage 2 of the growing season and 0.8 °C higher in stage 3 for 2048–2052. Temperature and precipitation in other stages of the growing season and time periods are within the historical range. Extrapolation to future climates substantially outside of the historical range would provide less reliable estimates. The future projections assume no change in management practices, cultivars, and technology, when in reality farmers are likely to adapt their practices to a changing climate. In addition, there are uncertainties in the input data such as historical temperature, precipitation and soil texture that could affect the estimation of yields. In terms of acceptability in diets, sorghum is culturally embedded as a staple in sorghum-growing regions, but wider acceptance would allow farmers to access a larger market.

In conclusion, a key measure for climate-smart agriculture is the promotion of C4 cereals in semi-arid and arid regions where farmers have traditionally grown these cereals. A focus on climate-smart agriculture for India’s *rabi* season highlights sorghum, the “camel of cereals”, as a nutritious, temperature-resilient and less water-demanding alternative to wheat if yields can be improved.

## Methods

### Historical patterns and trends

We identify patterns and trends in *rabi* cereal production with an updated annual time series from 1966 to 2017 of district-level crop area and production data^28^. The data separates *rabi* sorghum from *kharif* sorghum. Wheat is only a *rabi* crop. To harmonize analysis of the time series, we use the apportioned dataset with historical district boundaries available for 1961^29^ for all analyses. District boundaries have shifted and divided considerably over the time period. Currently, there are 741 districts, increasing from 324 in the dataset for 1961 boundaries.

### Sensitivity to historical and future temperature

To assess the historical temperature sensitivity of *rabi* cereal yields, we use a mixed-modeling framework following the approach of^20,30,31^. The analysis focuses on the 101 districts (historical boundaries) where both cereals were grown to maintain consistency in the range of climate conditions and other factors affecting yields. These common districts exclude the main wheat-growing region in northwest India and include the main area of sorghum cultivation in central India (Supplemental Fig. 1).

The two cereals differ in the timing of their growing seasons, which also vary in different locations (Supplemental Table 3)^32^. Excluding crop calendars from stations at Punjab and Himachal Pradesh, which are outside the 101 common districts, the first week of the wheat growing season varies among other stations from November 7 to Nov 21 and the last week from March 14 to April 11. For *rabi* sorghum, the first week varies from September 19 to October 3 and the last week from January 1 to February 14. Actual planting and harvesting dates can vary from these crop calendars. To include as much of the growing season as possible, for this analysis we consider the wheat growing season in the 101 common districts to be from November 7 to April 11 and the sorghum season to be September 19 to February 14. Sowing and emergence occur for sorghum in the first stage and for wheat in the second overlapping stage, while grain filling occurs in the overlapping stage for sorghum and the third stage for wheat.

From gridded maximum daily temperature at 1 × 1 degree resolution^33^ and precipitation data at 0.25 by 0.25 resolution^34^, we extract mean maximum temperature and precipitation for each district and each day between September 19 and April 11 for 1966–1967 to 2016–2017 (corresponding to harvest years 1966 and 2017 in the crop data). We obtain the median of the maximum daily temperature and the total precipitation for each district for three stages for each year: sorghum only from September 19 to November 6 (stage 1); overlapping sorghum and wheat from November 7 to February 13 (stage 2); and wheat only from February 14 to April 11 (stage 3). Statistics to obtain spatial means for districts were carried out with the R package “exactextractr”. We chose maximum daily temperature rather than mean or minimum based on wheat’s sensitivity to high temperatures.

Using only years in which both sorghum and wheat were produced in the district, sorghum yields were modeled as:

1 $$f\left({Y}_{s}\right)={\alpha sT}_{stage1}+ {\beta sT}_{stage2 }+ {\gamma sP}_{stage2} + {\delta sS}_{1}+ {\varepsilon sS}_{2}+\left(1|\text{d}\right)+\left(1|\text{t}\right),$$

and wheat yields were modeled as:

2 $$f\left({Y}_{w}\right)={\beta wT}_{stage2}+{\gamma wP}_{stage2 }+{\mu wT}_{stage3}+{\theta wP}_{stage3} +{\delta wS}_{1}+{\varepsilon wS}_{2}+\left(1|\text{d}\right)+\left(1|\text{t}\right),$$

where $${Y}_{s} \text{ and } {Y}_{w}=\text{sorghum and wheat yield respectively}$$; $${T}_{stage1 , }{T}_{stage2 , }\text{ and } {T}_{stage2 }=\text{median of maximum daily temperature in stages }1, 2,\text{ and }3;$$ $${P}_{stage2 }\text{ and } {T}_{stage2 }=\text{total precipitation in stages }2\text{ and }3;$$ $${S}_{1}\text{ and } {S}_{2}=\text{proportion of sand and silt},\text{ respectively};$$ $$\alpha s, \beta s, \gamma s, \delta s \text{ and } \varepsilon s \text{ are coefficients for the sorghum model};$$ $$\beta w, \gamma w, \mu w, \theta w, \delta w, \text{ and } \varepsilon w \text{ are coefficients for the wheat model}$$; and $$\left(1|\text{d}\right) \text{ and }\left(1|\text{t}\right) \text{represent random effects for district and time }\left(\text{year}\right)\text{ respectively}$$.

The random effect for year accounts for increases in yield over time due to management, inputs and cultivars, which is clearly evident in the data especially for wheat. We use the random effect for year rather than detrending the data due to incomplete time series for all districts and because the trends in yields are not linear. Inclusion of quadratic terms for precipitation and temperature did not improve the models so we included only the linear terms (Supplemental Table 4).

We do not include precipitation for stage 1 in the sorghum model because precipitation and temperature were co-linear for that stage (Supplemental Table 5). We also excluded proportion of clay soil due to co-linearity with proportions of sand and silt. Variance Inflation Factors for all variables are less than 2 for the models in Eqs. (1) and (2). Models were run with R package “lme4” using the “glmer” function. To determine p-value for the model coefficients, we used the “p_value” function in the “parameters” package.

To compare predicted and observed yields using alternative sources of climate data, we obtained historical climate data for 2010–2015 (the most recent available) from two models from the Climate Model Intercomparison Project (CMIP6) multi-ensemble model collection of models^35,36^. We selected these models because they are the only models from CMIP6 that provide all of the variables required for calculating crop water requirements. The models are the lower-complexity CNRM-CM6-1-HR at 0.25 by 0.25 degree resolution^37^ and higher-complexity, second generation CNRM-ESM2-1 at 1 by 1 degree resolution^38^. These models fall in the mid-range of climate sensitivity compared with other CMIP6 models^39^ and represent the Indian monsoon with reasonable accuracy (approximately within two standard deviations of historical rainfall)^40^.

Using the predict () function in R from the models and daily maximum temperatures and precipitation processed the same way as the climate data used to construct the models, we compared predicted and actual yields for 2010–2015. Adjusted R^2^ values for correlations between district-level actual and predicted yield values are between 0.55 and 0.87 from both sorghum and wheat models and root mean square errors (rmse) are between 0.084 and 0.182 (Supplemental Table 6). Because rmse values were generally lower using climate data from CNRM-ESM2-1 model, we show these results in the main text and results from the CNRM-CM6-1-HR model in Supplemental Material.

To explore the sensitivity of *rabi* sorghum and wheat yields to future climate, we again predicted yields based on the yield models from Eqs. (1) and (2) and maximum daily temperatures and precipitation obtained from the highest emissions Shared Socioeconomic Pathway (SSP5-8.5) from the climate models. The predicted yields were for the base time period (average for 2010–2015) and three future time periods: 5 year averages for 2028–2032, 2038–2042, and 2048–2052. We used the historical climate data provided with the climate models for the base time period rather than the data used to generate the model to ensure consistency in resolution and other factors that might lead to spurious results. The yields predicted from the models and climate data are intended to test sensitivity to possible future climate rather than to predict actual yield, which depends on management, varieties, pests, and many other factors in addition to climate.

Data sources are all publically available and are listed in Supplemental Table 7.

### Water requirements

Reference evapotranspiration for each of the stages (Sept 19–Nov 6; Nov 7–Feb 13; and Feb 14–April 11) was calculated for each of the time periods (2010–2015, 2028–2032, 2032–2042, 2048–2052) for each district. First we calculate monthly means for reference evapotranspiration using the United Nation’s Penman–Monteith equation^41^ with parameter values obtained from the CMIP-6 climate models:

3 $${ET}_{0}=\frac{0.408\Delta \left({R}_{n}-G\right)+\gamma \frac{900}{T+273}{u}_{2}\left({e}_{s}-{e}_{a}\right)}{\Delta +\gamma (1+0.34{u}_{2})},$$

where ${ET}_{0}$ = reference evapotranspiration (mm/day); ${R}_{n}$ = net radiation at the crop surface (MJ/m^2^/day); G = soil heat flux density (MJ/m^2^/day); T = air temperature at 2 m height (℃); ${u}_{2}$ = wind speed at 2 m height (m/s); ${e}_{s}$ = saturation vapour pressure (kPa); ${e}_{a}$ = actual vapour pressure (kPa); ${e}_{s}$-${e}_{a}$ = saturation vapour pressure deficit (kPa); $\Delta$ = slope vapour pressure curve (kPa/℃); and $\gamma$ = psychrometric constant (kPa/℃) (see Supplemental Table 8 for derivation of parameters).

Water requirements for wheat and sorghum in each stage were calculated from the reference evapotranspiration and crop coefficients and curves according to:

4 $${ET}_{c,j}=\sum_{i}^{Days}{ET}_{0,i}\times {k}_{c,i},$$

where ${ET}_{c,j}$= water requirement for crop *c* and crop development stage *j* and ${k}_{c,i}$ = crop coefficient for crop *c* and day *i* from Table 1 and Fig. 1 in Ref.^42^.

The crop water requirement (ETc) represents mm of water required to keep the plant from experiencing water stress. To estimate water footprints (volume of water required per unit of production in m^3^/tonne) we divided the crop water requirement for each district by yield obtained from the yield model in Eqs. (1) and (2).

## Supplementary Information

Supplementary Information.

## Data Availability

The datasets analyzed during the current study are publicly available in the data platforms listed in Supplemental Table 7.
