# Supplementary material for: Climate resilience of dry season cereals in India
Source: Sci Rep. 2023 Jun 20;13:9960. doi: 10.1038/s41598-023-37109-w (PMC10282032; doi:10.1038/s41598-023-37109-w)
Supplement: Supplementary file 1 — Supplementary Information. [file 41598_2023_37109_MOESM1_ESM.docx]

**SUPPLEMENTAL MATERIAL**

**CLIMATE RESILIENCE OF DRY SEASON CEREALS IN INDIA**

**Supplemental figure1**. Districts growing wheat, *rabi* sorghum, and both wheat and *rabi* sorghum in 2012-17. District boundaries are historical 1961 boundaries. Map was generated in QGiS 3.16.14.

**Supplemental figure 2.** Projections for daily maximum temperature averaged across time periods (2010-14; 2028-32; 2038-42; 2048-52) and common districts where both wheat and sorghum are grown from the CMIP6 CNRM-CM6-1-HR climate model.

**Supplemental figure 3**. Sensitivity of wheat yields to projections of maximum daily temperature from the CMIP6 CM6-HR-1 climate model in Supplemental figure 2. The sensitivity of wheat yields to maximum daily temperature is a difference in average yields across the districts by -0.10 ± 0.03 (sd), -0.15 ± 0.04, and -0.18 ± 0.04 tonnes/ha by 2028-32, 2038-42, and 2048-52 respectively relative to 2010-15 baseline in the common districts. These differences in yield correspond to percentage differences of -5.3 ± 1.7, -7.9 ± 2.0, and -9.7 ± 3.0 percent.

**Supplemental figure 4.** Same as Figure 6 with yield predictions and crop water requirements based on parameters from CMIP6 CM6-HR-1 climate model.

**Supplemental table 1.** Maximum daily temperature and total precipitation from the two climate models used in this study. Error ranges are 95% confidence intervals.

**Supplemental table 2.** Crop water requirement (mm) for baseline and future climate projections averaged for common districts for each stage using climate parameters from CNRM-ESM2-1 (top) and CNRM-CM6-1 (bottom). Error bars are 95% confidence interval.

**Supplemental table 3.** Crop calendar for stations throughout India from (*1*). We excluded Punjab and Himachal Pradesh sites from determining stages for models because they are outside of common wheat and sorghum districts

**Supplemental table 4.** R^2^ and Bayesian Information Criterion for sorghum and wheat models with linear terms only and with additional quadratic terms for temperature and precipitation. Lower BIC values for the same response variable indicate better fit.

**Supplemental table 5.** Correlation matrix for potential variables for sorghum and wheat models (top) and Variable Inflation Factors for models (bottom). Precipitation in stage 1 was eliminated from the sorghum model (not relevant for the wheat model because stage 1 is prior to the wheat growing season) and proportion of clay was eliminated from both models due to co-linearity. See text for variable abbreviations.

**Supplemental table 6.** Comparison of predicted with actual yields (tonnes/ha) for 2010-14 for districts with yield data available for both sorghum and wheat. 2010-14 are the years available for historical data from the climate models. All adjusted R^2^ values are significant at p<0.001. Top table is yield predictions with climate data from CNRM-ESM2-1 and bottom table is with CNRM-CM6-1.

**Supplemental table 7.** Sources for all data.

**Supplemental table 8**. Derivation for parameters in Penman-Monteith equation.

**Supplemental figure 1**. Districts growing wheat, *rabi* sorghum, and both wheat and *rabi* sorghum in 2012-17. District boundaries are historical 1961 boundaries.


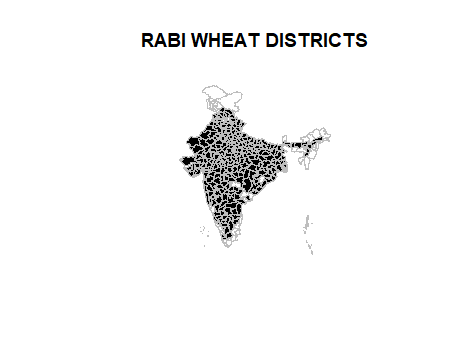


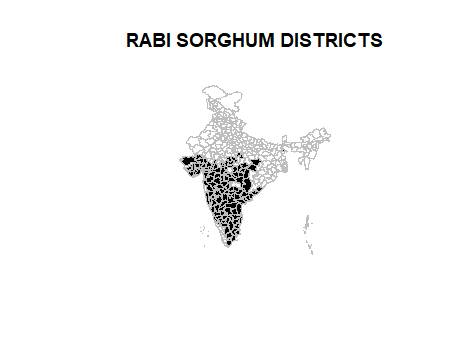


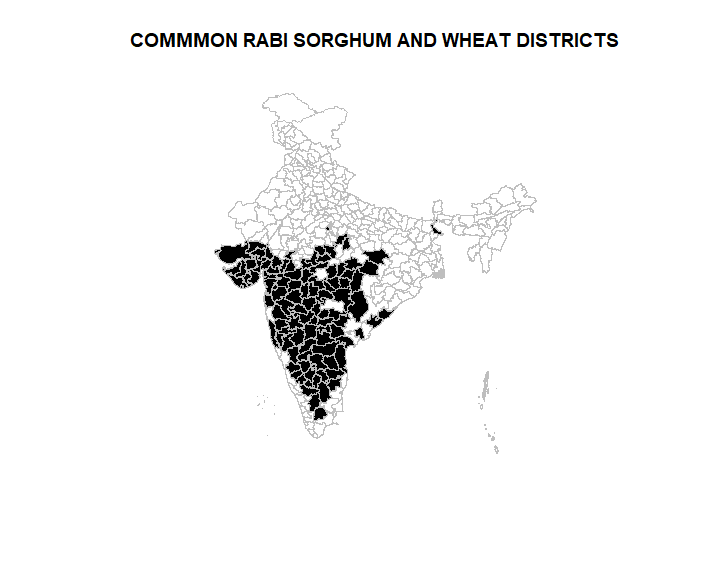


**Supplemental figure 2.** Projections for daily maximum temperature averaged across time periods (2010-14; 2028-32; 2038-42; 2048-52) and common districts where both wheat and sorghum are grown from the CMIP6 CNRM-CM6-1-HR climate model.


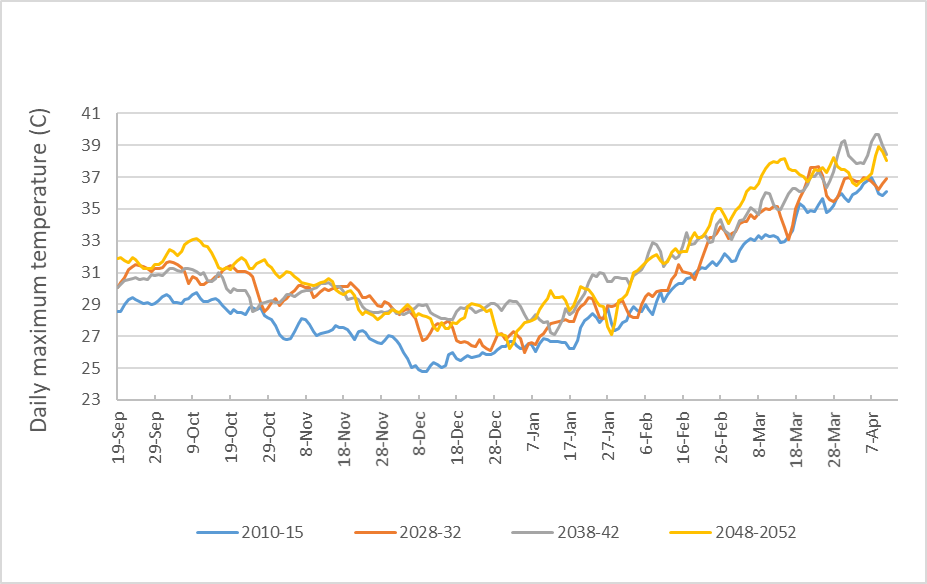


Wheat only (stage 3)

Sorghum and wheat (stage 2)

Sorghum only (stage 1)

**Supplemental figure 3.** Sensitivity of wheat yields to projections of maximum daily temperature from the CMIP6 CM6-HR-1 climate model in Supplemental figure “temp projections CM6-HR.” The sensitivity of wheat yields to maximum daily temperature is a difference in average yields across the districts by -0.10 ± 0.03 (sd), -0.15 ± 0.04, and -0.18 ± 0.04 tonnes/ha by 2028-32, 2038-42, and 2048-52 respectively relative to 2010-15 baseline in the common districts. These differences in yield correspond to percentage differences of -5.3 ± 1.7, -7.9 ± 2.0, and -9.7 ± 3.0 percent.

**
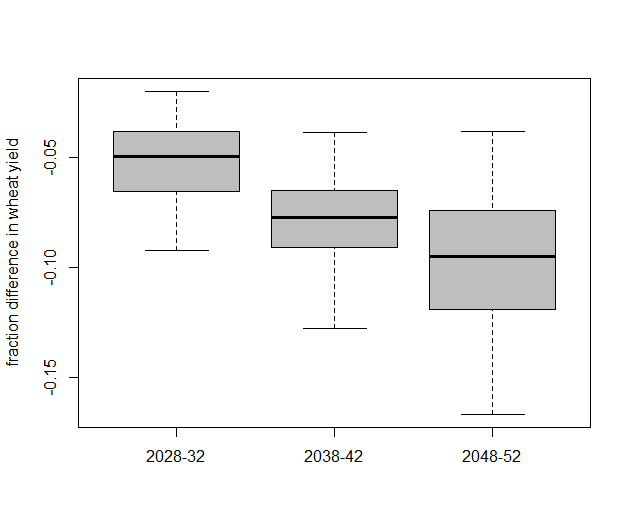
**
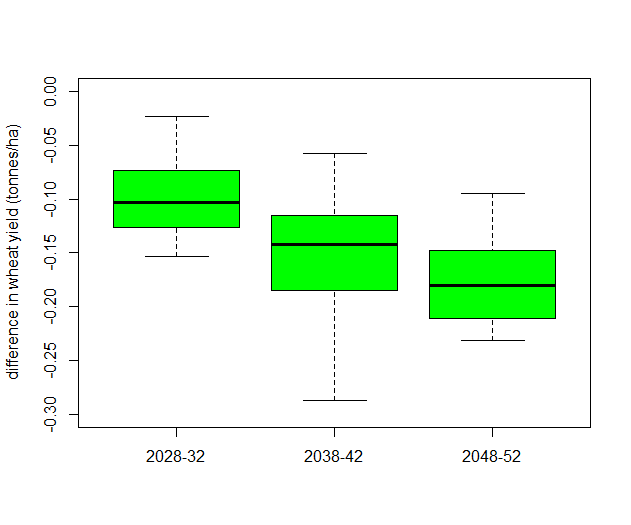


**Supplemental figure 4.** Same as Figure 6 with yield predictions and crop water requirements based on parameters from CMIP6 CM6-HR-1 climate model.


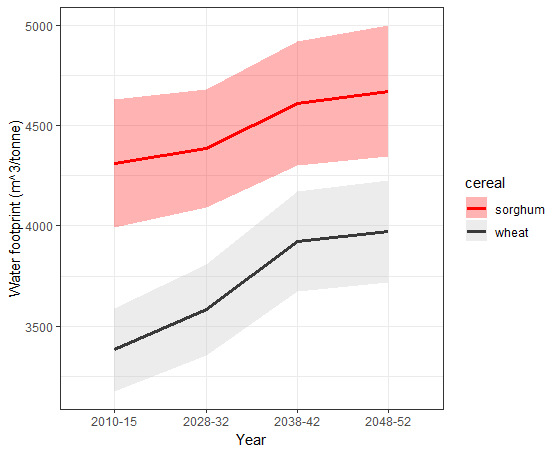


**Supplemental table 1.** Maximum daily temperature and total precipitation from the two climate models used in this study. Error ranges are 95% confidence intervals.

Maximum daily temperature (C) predicted from climate model averaged across common districts from CMIP6 CNRM-ESM2-1.

|  | MEDIAN OF MAX DAILY TEMPERATURE (C) FOR STAGE IN GROWING SEASON | | |
| --- | --- | --- | --- |
| TIME PERIOD | sorghum only  (Sept 19 to Nov 6) | sorghum and wheat (Nov 7 to Feb 13) | wheat only  (Feb 14 to April 11) |
| 2010-14 | 29.71 ± 0.23 | 27.91 ± 0.33 | 34.80 ± 0.32 |
| 2028-32 | 30.58 ± 0.28 | 28.53 ± 0.29 | 35.50 ± 0.34 |
| 2038-42 | 30.90 ± 0.36 | 28.95 ± 0.31 | 36.91 ± 0.36 |
| 2048-52 | 32.52 ± 0.42 | 30.63 ± 0.32 | 37.51 ± 0.34 |

Total precipitation (mm) predicted from climate model averaged across common districts from CMIP6 CNRM-ESM2-1 climate model.

|  | TOTAL PRECIPITATION (mm) FOR STAGE IN GROWING SEASON | | |
| --- | --- | --- | --- |
| TIME PERIOD | sorghum only  (Sept 19 to Nov 6) | sorghum and wheat (Nov 7 to Feb 13) | wheat only  (Feb 14 to April 11) |
| 2010-14 | 109.5 ± 13.8 | 45.6 ± 9.7 | 8.4 ± 2.2 |
| 2028-32 | 106.1 ± 15.3 | 59.2 ± 14.0 | 7.0 ± 1.5 |
| 2038-42 | 152.8 ± 25.1 | 49.5 ± 10.7 | 3.8 ± 1.8 |
| 2048-52 | 118.9 ± 23.2 | 38.7 ± 6.0 | 8.8 ± 3.9 |

Maximum daily temperature (C) predicted from climate model averaged across common districts from CMIP6 CM6-HR-1

|  | MEDIAN OF MAX DAILY TEMPERATURE (C) FOR STAGE IN GROWING SEASON | | |
| --- | --- | --- | --- |
| TIME PERIOD | sorghum only  (Sept 19 to Nov 6) | sorghum and wheat (Nov 7 to Feb 13) | wheat only  (Feb 14 to April 11) |
| 2010-14 | 28.89 ± 0.27 | 26.99 ± 0.40 | 33.68 ± 0.25 |
| 2028-32 | 30.82± 0.35 | 28.68 ± 0.35 | 34.93 ± 0.24 |
| 2038-42 | 30.41 ± 0.32 | 29.24 ± 0.28 | 35.85 ± 0.24 |
| 2048-52 | 31.84 ± 0.34 | 29.22 ± 0.37 | 36.43 ± 0.25 |

Total precipitation (mm) predicted from climate model averaged across common districts from CMIP6 CM6-HR-1

|  | TOTAL PRECIPITATION (mm) FOR STAGE IN GROWING SEASON | | |
| --- | --- | --- | --- |
| TIME PERIOD | sorghum only  (Sept 19 to Nov 6) | sorghum and wheat (Nov 7 to Feb 13) | wheat only  (Feb 14 to April 11) |
| 2010-14 | 125.3 ± 12.5 | 44.5 ± 6.1 | 8.1 ± 2.6 |
| 2028-32 | 85.0 ± 11.2 | 42.4 ± 8.3 | 7.4 ± 2.1 |
| 2038-42 | 126.5± 14.5 | 24.0 ± 6.2 | 9.7 ± 2.7 |
| 2048-52 | 100.4 ± 13.6 | 64.9 ± 12.1 | 11.6 ± 3.1 |

**Supplemental Table 2.** Crop water requirement (mm) for baseline and future climate projections averaged for common districts for each stage using climate parameters from CNRM-ESM2-1 (top) and CNRM-CM6-1 (bottom). Error bars are 95% confidence interval.

| CNRM-ESM2-1 |  | **STAGE OF GROWING SEASON** | | | |
| --- | --- | --- | --- | --- | --- |
|  |  | 19 SEPT-6 NOV  (49 days) | 7 NOV-13 FEB (99 days) | 14 FEB-APR 11  (57 days) | TOTAL |
| **TIME 1** (2010-15) | Median of max daily temperature (C) | 29.86 ± 0.23 | 28.14 ± 0.33 | 34.96 ± 0.32 |  |
|  | Sorghum cwr (mm) | 79.18±1.42 | 358.17±11.07 | -- | 437.35±12.26 |
|  | Wheat cwr (mm) | -- | 318.14±9.35 | 295.47±4.45 | 613.60±12.48 |
| **TIME 2** (2028-32) | Median of max daily temperature (C) | 30.80 ± 0.28 | 28.80 ± 0.29 | 35.59 ± 0.34 |  |
|  | Sorghum cwr (mm) | 82.63±1.17 | 383.03±9.84 | -- | 465.66±10.58 |
|  | Wheat cwr (mm) | -- | 346.57±8.39 | 321.64±5.41 | 668.8±12.60 |
| **TIME 3** (2038-42) | Median of max daily temperature (C) | 31.01 ± 0.36 | 29.34 ± 0.31 | 37.16 ± 0.36 |  |
|  | Sorghum cwr (mm) | 81.88±1.83 | 371.49±10.87 |  | 453.37±12.215 |
|  | Wheat cwr (mm) |  | 331.92±9.45 | 319.38±6.13 | 651.30±14.41 |
| **TIME 4** (2048-52) | Median of max daily temperature (C) | 32.73±0.42 | 31.07±0.32 | 37.96±0.34 |  |
|  | Sorghum cwr (mm) | 90.24±1.85 | 413.90±10.70 |  | 504.15±12.05 |
|  | Wheat cwr (mm) |  | 368.23±9.10 | 326.93±6.49 | 695.15±14.36 |

| CNRM-CM6-1 |  | **STAGE OF GROWING SEASON** | | | |
| --- | --- | --- | --- | --- | --- |
|  |  | 19 SEPT-6 NOV  (49 days) | 7 NOV-13 FEB (99 days) | 14 FEB-APR 11  (57 days) | TOTAL |
| **TIME 1** (2010-14) | Median of max daily temperature (C) | 28.89± 0.27 | 26.99 ± 0.40 | 33.68 ± 0.25 |  |
|  | Sorghum cwr (mm) | 77.33±1.03 | 361.32±9.33 | -- | 438.64±9.99 |
|  | Wheat cwr (mm) | -- | 321.44±7.75 | 289.24±4.41 | 610.68±10.51 |
| **TIME 2** (2028-32) | Median of max daily temperature (C) | 30.82± 0.35 | 28.68 ± 0.35 | 35.93 ± 0.24 |  |
|  | Sorghum cwr (mm) | 85.31±1.83 | 363.75±10.54 | -- | 449.05±11.88 |
|  | Wheat cwr (mm) | -- | 319.94±8.78 | 290.06±4.91 | 610.00±12.65 |
| **TIME 3** (2038-42) | Median of max daily temperature (C) | 30.41 ± 0.32 | 29.28 ± 0.28 | 35.85 ± 0.24 |  |
|  | Sorghum cwr (mm) | 80.66±1.40 | 390.39±9.40 |  | 471.06±10.33 |
|  | Wheat cwr (mm) |  | 344.02±7.96 | 303.30±5.38 | 647.32±11.98 |
| **TIME 4** (2048-52) | Median of max daily temperature (C) | 31.84±0.34 | 29.22±0.37 | 36.43±0.25 |  |
|  | Sorghum cwr (mm) | 88.25±1.41 | 387.12±8.93 |  | 475.38±9.80 |
|  | Wheat cwr (mm) |  | 339.41±7.48 | 302.49±4.24 | 641.89±10.20 |

**Supplemental table 3.** Crop calendar for stations throughout India from (*1*). We excluded Punjab and Himachal Pradesh sites from determining stages for models because they are outside of common wheat and sorghum districts

| **SITE** | **CROP** | **BEGINNING WEEK** | **END WEEK** |
| --- | --- | --- | --- |
| Ludhiana, Punjab | WHEAT | 24-Oct | 11-Apr |
| Anand, Gujurat | WHEAT | 14-Nov | 14-Mar |
| Palampur, Himachal Pradesh | WHEAT | 17-Oct | 9-May |
| Raipur, Chattisgarh | WHEAT | 21-Nov | 11-Apr |
| Udaipur, Rajasthan | WHEAT | 7-Nov | 4-Apr |
| Ranchi, Jharkhand | WHEAT | 21-Nov | 28-Mar |
| Bijapur, Karnataka | RABI SORGHUM | 19-Sep | 14-Feb |
| Kovilpattu, Tamilnadu | RABI SORGHUM | 3-Oct | 1-Jan |
| Solapur, Maharashtra | RABI SORGHUM | 3-Oct | 31-Jan |

| **DEFINITIONS OF STAGES FOR THIS ANALYSIS** | | |
| --- | --- | --- |
|  | START DATE | END DATE |
| WHEAT GROWING SEASON | 07-Nov | 11-Apr |
| RABI SORGHUM GROWING SEASON | 19-Sep | 14-Feb |
| STAGE 1: SORGHUM PLANTING PRIOR TO WHEAT PLANTINGCOMMON GROWING SEASON | 19-Sep | 06-Nov |
| STAGE 2: OVERLAPPING GROWING SEASON FOR WHEAT AND SORGHUM | 07-Nov | 13-Feb |
| STAGE 3: POST-HARVEST FOR SORGHUM AND WITHIN WHEAT GROWING SEASON | 14-Feb | 11-Apr |

**Supplemental table 4.** R^2^ and Bayesian Information Criterion for sorghum and wheat models with linear terms only and with additional quadratic terms for temperature and precipitation. Lower BIC values for the same response variable indicate better fit.

| **Cereal** | **sorghum** | **sorghum** | **wheat** | **wheat** |
| --- | --- | --- | --- | --- |
| **Model** | linear | quadratic | linear | quadratic |
| **BIC** | 40956 | 40965 | 41090 | 41102 |
| **Conditional R^2^** | 0.74 | 0.74 | 0.79 | 0.79 |
| **Marginal R^2^** | 0.09 | 0.09 | 0.03 | 0.03 |

**Supplemental table 5**. Correlation matrix for potential variables for sorghum and wheat models (top) and Variable Inflation Factors for models (bottom). Precipitation in stage 1 was eliminated from the sorghum model (not relevant for the wheat model because stage 1 is prior to the wheat growing season) and proportion of clay was eliminated from both models due to co-linearity. See text for variable abbreviations.

|  | T_stage1_ | P_stage1_ | T_stage2_ | P_stage2_ | T_stage3_ | P_stage3_ | S_0 (clay)_ | S_1 (sand)_ | S_2 (silt)_ |
| --- | --- | --- | --- | --- | --- | --- | --- | --- | --- |
| T_stage1_ | 1.00 |  |  |  |  |  |  |  |  |
| P_stage1_ | **-0.69** | 1.00 |  |  |  |  |  |  |  |
| T_stage2_ | 0.23 | -0.07 | 1.00 |  |  |  |  |  |  |
| P_stage2_ | -0.14 | 0.12 | -0.24 | 1.00 |  |  |  |  |  |
| T_stage3_ | 0.13 | 0.00 | 0.39 | -0.12 | 1.00 |  |  |  |  |
| P_stage3_ | -0.23 | 0.10 | -0.06 | 0.16 | -0.12 | 1.00 |  |  |  |
| S_0 (clay)_ | -0.34 | 0.22 | -0.26 | 0.17 | -0.40 | 0.04 | 1.00 |  |  |
| S_1 (sand)_ | 0.46 | -0.26 | 0.28 | -0.24 | 0.31 | -0.10 | **-0.79** | 1.00 |  |
| S_2 (silt)_ | -0.01 | -0.03 | 0.08 | 0.01 | 0.26 | 0.06 | **-0.65** | 0.05 | 1.00 |

| **model** | **wheat** | **sorghum** |
| --- | --- | --- |
| Variables: |  |  |
| T_stage1_ | -- | 1.21 |
| P_stage1_ | -- | -- |
| T_stage2_ | 1.12 | 1.26 |
| P_stage2_ | 1.10 | 1.25 |
| T_stage3_ | 1.18 |  |
| P_stage3_ | 1.14 | -- |
| S_1 (sand)_ | 1.02 | 1.05 |
| S_2 (silt)_ | 1.01 | 1.01 |

**Supplemental table 6.** Comparison of predicted with actual yields (tonnes/ha) for 2010-14 for districts with yield data available for both sorghum and wheat. 2010-14 are the years available for historical data from the climate models. All adjusted R^2^ values are significant at p<0.001. Top table is yield predictions with climate data from CNRM-ESM2-1 and bottom table is with CNRM-CM6-1.

| CNRM-ESM2-1 | 2010 | 2011 | 2012 | 2013 | 2014 |
| --- | --- | --- | --- | --- | --- |
| WHEAT: |  |  |  |  |  |
| rmse | 0.094 | 0.056 | 0.061 | 0.102 | 0.059 |
| adjusted r^2^ | 0.80 | 0.83 | 0.79 | 0.87 | 0.78 |
| average actual yield | 1.880±0.153 | 1.900±0.166 | 1.787±0.176 | 1.745±0.168 | 1.582±0.154 |
| average predicted yield | 1.974±0.117 | 1.957±0.116 | 1.848±0.109 | 1.847±0.118 | 1.642±0.119 |
| SORGHUM: |  |  |  |  |  |
| rmse | 0.105 | 0.084 | 0.116 | 0.099 | 0.079 |
| adjusted r^2^ | 0.57 | 0.69 | 0.69 | 0.63 | 0.72 |
| average actual yield | 1.169±0.133 | 1.006±0.104 | 1.095±0.156 | 1.059±0.086 | 0.989±0.083 |
| average predicted yield | 1.064±0.062 | 0.919±0.104 | 0.979±0.051 | 0.957±0.047 | 0.910±0.040 |
| number of districts | 64 | 56 | 50 | 50 | 44 |

| CNRM-CM6-1 | 2010 | 2011 | 2012 | 2013 | 2014 |
| --- | --- | --- | --- | --- | --- |
| WHEAT: |  |  |  |  |  |
| rmse | 0.182 | 0.140 | 0.150 | 0.173 | 0.137 |
| adjusted r^2^ | 0.80 | 0.84 | 0.80 | 0.87 | 0.75 |
| average actual yield | 1.880±0.153 | 1.900±0.166 | 1.787±0.176 | 1.745±0.168 | 1.582±0.154 |
| average predicted yield | 2.062±0.119 | 2.040±0.118 | 1.933±0.110 | 1.918±0.118 | 1.720±0.118 |
| SORGHUM: |  |  |  |  |  |
| rmse | 0.134 | 0.114 | 0.147 | 0.131 | 0.107 |
| adjusted r^2^ | 0.55 | 0.67 | 0.67 | 0.61 | 0.70 |
| average actual yield | 1.169±0.133 | 1.006±0.104 | 1.095±0.156 | 1.059±0.086 | 0.989±0.083 |
| average predicted yield | 1.037±0.062 | 0.892±0.050 | 0.948±0.052 | 0.929±0.049 | 0.882±0.042 |
| number of districts | 64 | 56 | 50 | 50 | 44 |

**Supplemental table 7.** Sources for all data.

| **DATA** | **CITATION** | **AVAILABLE FROM** | **USE** |
| --- | --- | --- | --- |
| Historical crop data | (*2*) | http://vdsa.icrisat.ac.in/ | response variable for yield model |
| Historical max daily temperature | (*3*) | https://www.imdpune.gov.in/Clim_Pred_LRF_New/Grided_Data_Download.html | predictor variable for yield model |
| Historical daily precipitation | (*4*) | https://www.imdpune.gov.in/Clim_Pred_LRF_New/Grided_Data_Download.html | Covariate for yield model |
| Soil texture | (*5*) | https://www.isric.org/explore/wise-databases | Covariate for yield model |
| Future and 2010-14 max daily temperature; precipitation; and parameters for reference evapotranspiration | (*6, 7*) | https://cds.climate.copernicus.eu/ | reference ET parameters and future projections |
| Crop calendar | (*1*) |  | growing season stages |
| District boundaries for 1961 | (*8*) | https://geodata.mit.edu/catalog/sde-columbia-mlinfomapdistrict61 | district zonal stats |

**Supplemental table 8.** Derivation for parameters in Penman-Monteith equation

For $R_{n}$:

$R_{n}=R_{ns}- R_{nl}=\left( R_{sd}-R_{su} \right)-\left( R_{lu}-R_{ld} \right)$ (1)

Where

$R_{ns}$: net surface shortwave radiation (MJ/m^2^/day)

$R_{nl}$: net surface longwave radiation (MJ/m^2^/day)

$R_{sd}$: surface downwelling shortwave radiation (MJ/m^2^/day)

$R_{su}$: surface upwelling shortwave radiation (MJ/m^2^/day)

$R_{lu}$: surface upwelling longwave radiation (MJ/m^2^/day)

$R_{ld}$: surface downwelling longwave radiation (MJ/m^2^/day)

For G:

For daily or ten-day step, G ≈ 0;

For monthly,

$G=0.14\times\left( T_{month,i}-T_{month,i-1} \right)$ (2)

For T:

$T=\frac{T_{max}-T_{min}}{2}$ (3)

For wind speed at 2 m height:

If we have the wind speed at z m height, we need convert it to wind speed at 2 m height.

$u_{2}=u_{z}\frac{4.87}{ln\left( 67.7z-5.42 \right)}$ (4)

Where

$u_{z}$: wind speed at z m height (m/s)

For $e_{s}$ and $e_{a}$:

$e^{0}\left( T \right)=0.6108exp\left( \frac{17.27T}{T+237.3} \right)$ (5)

$e_{s}=\frac{e^{0}\left( T_{max} \right)+e^{0}\left( T_{min} \right)}{2}$ (6)

$e_{a}=e^{0}\left( T_{dew} \right)$ (7)

Where

$T_{dew}$: dewpoint temperature at 2 m (℃)

For $\Delta$:

$\Delta=\frac{4098\left[ 0.6108exp\left( \frac{17.27T}{T+237.3} \right) \right]}{\left( T+237.3 \right)^{2}}$ (8)

For $\gamma$:

$\gamma=0.665\times{10}^{-3}\times P$ (9)

Where

$P$: atmospheric pressure (kPa)

REFERENCES

1. V. Rao *et al.*, District level crop weather calendars of major crops in India. (2015).

2. International Crops Research Institute for the Semi-Arid Tropics, "Village Dynamics in South Asia meo level data for India: 1966-2011," (ICRISAT, Hyderabad, 2015).

3. A. Srivastava, M. Rajeevan, S. Kshirsagar, Development of a high resolution daily gridded temperature data set (1969–2005) for the Indian region. *Atmospheric Science Letters* **10**, 249-254 (2009).

4. M. Rajeevan, J. Bhate, J. Kale, B. Lal, High resolution daily gridded rainfall data for the Indian region: Analysis of break and active monsoon spells. *Current Science* **91**, 296-306 (2006).

5. N. H. Batjes, Harmonized soil property values for broad-scale modelling (WISE30sec) with estimates of global soil carbon stocks. *Geoderma* **269**, 61-68 (2016).

6. C. Tebaldi *et al.*, Climate model projections from the scenario model intercomparison project (ScenarioMIP) of CMIP6. *Earth System Dynamics* **12**, 253-293 (2021).

7. A. Voldoire *et al.*, Evaluation of CMIP6 deck experiments with CNRM‐CM6‐1. *Journal of Advances in Modeling Earth Systems* **11**, 2177-2213 (2019).

8. ML InfoMap. (ML InfoMap Pvt. Ltd., 1961).
